# Gleanings from the French

**Published:** 1874-12-15

**Authors:** 


### GLEANINGS FROM THE FRENCH.

*Translated by Fred. J. Huse, M.D.*

UNCIPRESSURE. — A new method for controlling traumatic hæmorrhages in certain cases where circumstances of time and place, or other considerations, may render ligation of the arteries inefficient, has been suggested by Dr. Vanzetti, of Padua, in a communication to the Surgical Society of Paris. He cites three instances in which he has made successful use of this method.

The first is that of a robust peasant, fifty years of age, who had received a cut across the back of his hand in the first intermetacarpal space. The hæmorrhage was at first controlled by a compressive bandage, but thirteen days after the accident a formidable hæmorrhage suddenly set in, and continuing for several days, in spite of the compression, the patient was brought to Dr. Vanzetti. The wound was nearly two inches in length; the hand was much swollen. The surgeon sought the artery in the centre of the suppurating wound, believed that he found it, applied ligatures, and the blood ceased to flow.

Nevertheless, not being certain that he had carried the silk around the wounded artery, Dr. Vanzetti made use as complement of the hæmostasis, of the following method: He held down the lips of the wound by means of two hooks forced deeply into the edge of each flap, and maintained the pressure by attaching them to the bed-slats. The third day, one of the margins showing signs of returning hæmorrhage, the hook of that side was placed over the point of the appearance of the blood; the hæmorrhage then ceased. Subsequently the same accident was treated in the same manner on the other side of the wound. The hooks were removed forty hours after application and the patient recovered.

The two other cases mentioned in this article of Dr. Vanzetti, are instances of the application of hooks at the beginning of hæmorrhage. The pressure of the hook upon an artery flattens its calibre and changes the relations of the vessel with the surrounding tissues. The hooks may have different shapes and may be applied in different manners, while they should be left in position for a longer or shorter time, according to the volume of the artery. The wounded region requires to be immobilized in order to maintain the tension of the hooks upon the borders of the wound.

---

The Gazette Medicale, of Bordeaux, mentions a report to the medical society of that city, by a veterinary surgeon, concerning the practice among coachmen belonging to certain well-to-do bourgeois families, of administering chloral to the horses under their care for the purpose of rendering them less restive, and accordingly more easily driven. The chloral is said to work like a charm, so that horses previously held in with the greatest difficulty, after a few days of this hyposthenic regimen, become most quiet and docile, as well as lazy and sleepy.

---

Action of Ipecac in Diarrhœa.—After much careful experimentation, Dr. Polichronic has announced that ipecac serves to provoke an intense inflammation of the mucous membrane of the intestinal canal. By thus replacing the spurious phlegmasia which exists in chronic diarrhœa, ipecac promotes the speedy and spontaneous recovery of such cases.

---

A New Method of Extraction of Cataract.—Dr. R. Castorani is announced in *La France Medicale* as having lately presented to the consideration of the Parisian Academy of Sciences, a new method for the external linear extraction of cataract. The principal novelty of this method consists in the opening of the cornea, or sclerotica, to the extent of four or five lines, by a simple puncture of a broad, curved keratotome. Iridectomy is then performed and the lens is extracted within the capsule. Dr. C. has specially adapted all the instruments necessary to the successive stages of the operation.

The principal advantage of this method consists in the easy adaptation and reunion of the margins of the wound even when all the vitreous humor has been removed. Reunion after linear puncture is much easier than after linear section, and chiefly by reason of the action of the lids in making pressure. The aqueous humor is also more completely retained by this method, for the same reason, and it is claimed that there is thus obviated any danger of interocular hæmorrhage after removal of the vitreous humor, as the continual flow of the aqueous humor fills up the ocular cavity and preserves its natural form and volume.

---

The Drs. Mayencon and Bergeret have announced in *La France Medicale*, as the result of extended observations in regard to the action of arsenic and antimony upon the organisms of men and animals, the following general conclusions:

1. Arsenic is absorbed and diffused in the organism with very great promptness. Elimination through the urine takes place immediately.

2. Antimony is absorbed and diffused more slowly. Urinary elimination rarely begins on the first day.

3. Arsenic is eliminated simultaneously by the liver and kidneys, but more by the liver than by the kidneys.

4. Antimony is carried off in a much larger quantity by the liver than by the kidneys.

